# Complex CTO Revascularization in Patients with Ischemic Heart Failure and Reduced Ejection Fraction: An Illustrative Case Series

**DOI:** 10.3390/jcm15114235

**Published:** 2026-05-30

**Authors:** Ioana Paula Blaj-Tunduc, Mihnea-Traian Nichita-Brendea, Vlad-Victor Babes, Ioana Adela Ratiu, Emilia Elena Babeș

**Affiliations:** 1 Doctoral School of Biomedical Sciences, Faculty of Medicine and Pharmacy, University of Oradea, 410087 Oradea, Romania; blaj.ioanapaula@student.uoradea.ro (I.P.B.-T.); eebabes@uoradea.ro (E.E.B.); 2 Cardiology Department, Emergency Clinical Hospital, Bihor County, 65 Gheorghe Doja Street, 410169 Oradea, Romania; niki_napo@yahoo.com; 3 Department of Medical Disciplines, Faculty of Medicine and Pharmacy, University of Oradea, 410073 Oradea, Romania; 4 Nephrology Department, Emergency Clinical Hospital, Bihor County, 12 Corneliu Coposu Street, 410469 Oradea, Romania

**Keywords:** chronic total occlusion, heart failure, viability, percutaneous coronary intervention

## Abstract

**Background/Objectives**: Revascularization of chronic total occlusions (CTO) in patients with heart failure and reduced ejection fraction (HFrEF) remains controversial, as randomized trials have not demonstrated a clear prognostic benefit. **Methods**: We present an imaging-guided case series of patients with ischemic HFrEF who underwent CTO percutaneous coronary intervention (PCI) following myocardial viability assessment using single-photon emission computed tomography (SPECT). Contemporary antegrade and retrograde techniques were employed. **Results**: At 6- and 12-month follow-ups, all patients demonstrated marked improvement in NYHA (New York Heart Association) functional class, significant reductions in NT-proBNP (N-terminal pro-brain natriuretic peptide) levels, and substantial improvement in quality of life assessed by the Minnesota Living with Heart Failure Questionnaire (MLHFQ). These benefits occurred despite only modest improvement in left ventricular (LV) ejection fraction (EF) and limited reverse remodeling. SPECT enabled identification of viable but ischemic myocardium, supporting individualized revascularization decisions. **Conclusions**: In selected high-risk patients with ischemic HFrEF, CTO-PCI was associated with meaningful clinical and biomarker improvement independent of substantial EF recovery. Careful patient selection, incorporating myocardial viability assessment, may refine individualized clinical decision-making in selected patients. These findings support an imaging-guided approach and warrant further prospective evaluation.

## 1. Introduction

Chronic total occlusion (CTO) remains one of the most challenging and complex subsets of coronary artery disease (CAD), discovered in almost 20% of patients with chronic coronary syndrome (CCS) [1,2]. CTOs are usually associated with heart failure (HF) symptoms, angina, adverse remodeling of the left ventricle (LV) with systolic and/or diastolic dysfunction, and impaired quality of life, which can increase cardiovascular risk and restrict exercise capacity [2,3]. Especially in patients with HFrEF, CTO remains a high-risk substrate that maintains ongoing ischemia and adds a worse prognosis with recurrent decompensation [1,4].

Despite significant advances in contemporary CTO-PCI intervention technique, randomized trials such as REVASC (Regional Left Ventricular Function after Stent Implantation in Chronic Total Occlusion), EXPLORE-CTO (Evaluating Xience and Left Ventricular Function in PCI on Occlusions After STEMI), DECISION-CTO (Optimal Medical Therapy With or Without Stenting for Coronary Chronic Total Occlusion), and EURO-CTO (The Randomized Multicenter Trial to Compare Revascularization With Optimal Medical Therapy for the Treatment of Chronic Total Occlusions) failed to demonstrate clear benefit regarding mortality, major adverse cardiovascular events, or LV functional recovery compared with conservative treatment [5,6,7,8]. Consequently, the American College of Cardiology/American Heart Association/Society for Cardiovascular Angiography and Interventions 2021 coronary revascularization Guidelines downgraded CTO-PCI recommendations from class IIA to IIB [9]. Important limitations of these trials were the underrepresentation of high-risk patients (particularly those with multiple comorbidities or severely reduced EF) and the lack of myocardial viability assessment in guiding patient selection for revascularization [10].

Myocardial perfusion SPECT remains one of the most widely used imaging modalities for myocardial viability assessment of ischemic cardiomyopathy. By identifying viable but ischemic myocardium, SPECT may help select patients who could derive symptomatic and functional benefit from revascularization, even in the absence of substantial recovery of global systolic function [11,12,13,14,15]. Therefore, in clinical practice, the decision to perform CTO-PCI should be individualized and based on multiple factors, including the patient’s clinical status, coronary anatomy, procedural complexity, LV function, and multimodality imaging within a multidisciplinary Heart Team approach [9,16,17,18,19,20].

Importantly, in patients with ischemic HFrEF, optimal medical therapy (OMT) remains the cornerstone of management, with guideline-directed medical therapy (GDMT) recommended as first-line treatment to improve survival and reduce hospitalizations [21,22]. Revascularization decisions should therefore be made within the Heart Team framework, on top of comprehensive OMT, tailored to the individual patient’s clinical status and coronary anatomy.

In this context, we present three selected cases of patients with symptomatic ischemic HFrEF despite OMT, in whom CTO-PCI was performed following SPECT demonstration of myocardial viability. These cases highlight the potential role of imaging-guided patient selection and suggest that CTO-PCI may provide meaningful clinical and functional benefit in carefully chosen high-risk patients.

## 2. Materials and Methods

This study represents an imaging-guided case series of patients with ischemic HFrEF who each presented with at least one CTO and underwent PCI between 2024 and 2025 at the Clinical Emergency County Hospital Bihor in Oradea, Romania. The presented cases were selected to illustrate the clinical course and imaging findings of patients undergoing viability-guided CTO-PCI.

### 2.1. Inclusion Criteria

The inclusion criteria were the presence of at least one angiographically confirmed CTO (TIMI 0 flow for ≥3 months), class NYHA III-IV HF symptoms or refractory angina despite OMT, reduced left ventricular EF < 40%, and the presence of viable myocardium in the territory supplied by CTO. Patients were included consecutively when all criteria were met, and PCI was considered clinically indicated based on symptoms and imaging findings.

### 2.2. Imaging Protocol and Viability Assessment

Before revascularization, all patients underwent a comprehensive clinical and paraclinical evaluation. Transthoracic echocardiography was performed using a Vivid E 95 ultrasound system (GE Vingmed Ultrasound AS, Strandpromenaden 45, 3191 Horten, Norway). Coronary angiography and CTO-PCI procedures were conducted using a Philips angiography system (Philips Medical System Nederland B.V. Venpluis 4-6 Best, The Netherlands). Pharmacologic stress myocardial perfusion single-photon emission computed tomography (SPECT) was achieved using a dual-detector E.cam gamma camera (Siemens Medical System Nuclear Medicine Group, Hoffman Estates, IL, USA), and image processing and quantitative analysis were accomplished using Xeleris software version 2.27 (GE HealthCare, Chicago, IL, USA). Myocardial perfusion SPECT was performed using a vasodilator stress (adenosine). Stress and rest acquisitions were carried out following the institutional imaging protocol to assess myocardial viability and the presence of inducible ischemia. All acquisitions were obtained using ECG-gated SPECT with standard short-axis, vertical long-axis, and horizontal long-axis reconstructions. Rest imaging enabled differentiation between viable and scarred myocardium; in contrast, stress imaging identified reversible ischemia in affected segments. Reversible defects were interpreted as inducible ischemia and fixed defects as scarred myocardium. Myocardial segments were considered viable when radiotracer uptake exceeded 50% of the maximal regional uptake.

Patients were considered appropriate for CTO-PCI when SPECT showed viable myocardium within the CTO-related territory (defined as preserved radiotracer uptake >50%) associated with reversible ischemia on stress-rest imaging, in the presence of persistent symptoms despite OMT. Decisions regarding revascularization were reached through a multidisciplinary Heart Team that integrated imaging findings, clinical status, coronary anatomy, and procedural feasibility.

### 2.3. PCI Procedure and Lesion Assessment

The severity and complexity of CTO were graded using the J-CTO score [23]. PCI was performed according to the operator’s judgement and lesion characteristics by approaching the lesion with contemporary anterograde and/or retrograde techniques. Procedural success was defined as restoration of coronary blood flow with Thrombolysis in Myocardial Infarction (TIMI) grade 3, adequate myocardial blush grade (MBG ≥ 2), and successful stent implantation without in-hospital major complications [24,25,26].

### 2.4. Clinical and Functional Assessment

Clinical status and paraclinical investigation (echocardiography, biomarkers) were evaluated at baseline, 6 months, and 12 months after CTO-PCI. To assess the quality of life before and after revascularization, we used the Minnesota Living with Heart Failure Questionnaire (MLHFQ), with scores ranging from 0 to 105 (higher scores meaning poorer quality of life) [27].

The primary endpoints were changes in NYHA functional class, MLHFQ score, and NT-proBNP levels. Secondary endpoints included changes in LVEF and volumes, as well as the incidence of HF rehospitalizations and periprocedural complications.

All the ethical requirements regarding patients were respected, and informed consent was obtained from all subjects involved, allowing the use of their data in future publications. The study was performed in accordance with the Declaration of Helsinki and approved by the Ethics Committee for Scientific Research of Clinical Emergency Bihor County Hospital, Oradea, Romania. (Decision 34171/7 November 2024).

## 3. Case Presentation

The three illustrative cases of CTO revascularization in patients with reduced EF are presented below, focusing on clinical presentation, procedural strategy, and functional recovery, all individually and in a comparative manner, to enhance interpretability.

### 3.1. Case 1

A 74-year-old woman from a rural area, a former smoker with a 15-year history of diabetes mellitus, arterial hypertension, and dyslipidemia, and known multivessel coronary artery disease treated with PCI, presented to the emergency department with unstable angina and exertional dyspnea. Her coronary history included the placement of two bare-metal stents in the right coronary artery (RCA) and two in the circumflex artery (LCx) in 2008, following an inferior myocardial infarction. In 2019, she developed in-stent restenosis in both vessels along with 50–75% stenosis of the left anterior descending artery (LAD) ostium, for which she underwent complex multivessel PCI with drug-eluting stents to the RCA, LCx, and LAD. Despite revascularization, she experienced progressive clinical deterioration and recurrent episodes of unstable angina between 2020 and 2024. The baseline Minnesota Living with Heart Failure Questionnaire (MLHFQ) score was 56.

Electrocardiography showed inferior Q waves consistent with prior myocardial infarction (2008). Transthoracic echocardiography (TTE) revealed a dilated left ventricle with reduced systolic function (LVEF 37%), impaired LV relaxation (E/A ratio less than 1, prolonged DT (deceleration time), mildly prolonged IVRT (isovolumic relaxation time)), and mild-to-moderate functional mitral regurgitation (Table 1). Laboratory testing demonstrated preserved renal function (creatinine 0.89 mg/dL), optimally controlled LDL cholesterol (47 mg/dL), elevated NT-proBNP (3570 pg/mL), normal high-sensitivity troponin, and normoglycemia (fasting glucose 101 mg/dL). At presentation, she was receiving guideline-directed medical therapy for HF and ischemic heart disease, including dual antiplatelet therapy (aspirin and clopidogrel), a beta-blocker (bisoprolol), ARNI (Angiotensin Receptor–Neprilysin Inhibitor), a mineralocorticoid receptor antagonist (spironolactone), SGLT2i (sodium–glucose cotransporter-2 inhibitors), a loop diuretic (Furosemide), and intensive lipid-lowering therapy (atorvastatin plus ezetimibe).

**Figure 1:**
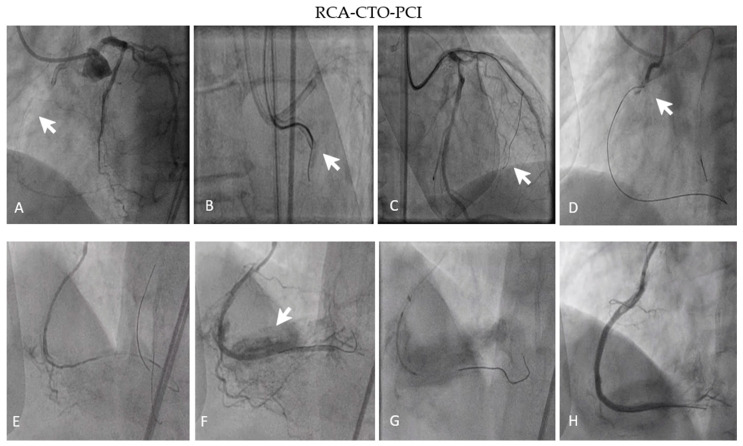
Percutaneous revascularization of a right coronary artery total chronic occlusion. Legend: RCA—right coronary artery, CTO—chronic total occlusion, PCI—percutaneous coronary intervention. (**A**) Diagnostic coronary angiography, CTO of RCA (white arrow); (**B**) unsuccessful antegrade crossing attempt (white arrow); (**C**) Approaching septal collaterals (white arrow); (**D**) intentional antegrade dissection (white arrow) to get subintimal; (**E**) Reverse Controlled Antegrade and Retrograde Tracking/Tip-in connection; (**F**,**G**) compressive hematoma (white arrow) in the distal part of the RCA; (**H**) final result.

Diagnostic coronary angiography revealed patent stents on the LAD and LCx and CTO of the RCA (Figure 1). We performed a SPECT study to assess myocardial viability. The imaging procedure demonstrated preserved tracer uptake in the territory supplied by the RCA, suggesting viable myocardium and inducible ischemia, findings potentially supportive of revascularization. Based on the patient’s persistent symptoms and the SPECT-documented viability, CTO-PCI of the RCA was performed.

**Figure 2:**
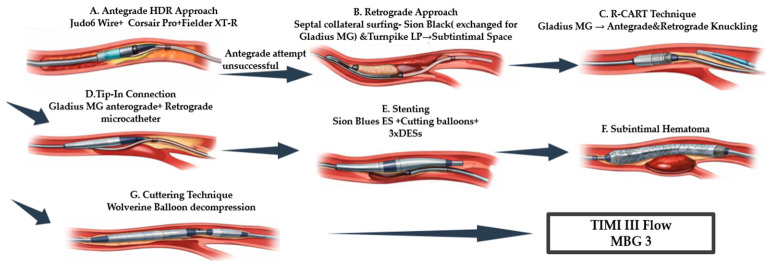
PCI-RCA-CTO step by step. Legend: HDR—hydrodynamic plaque recanalization, R-CART—Reverse Controlled Antegrade and Retrograde Tracking, TIMI—Thrombolysis in Myocardial Infarction, MBG—Myocardial Blush Grade.

We approached the RCA-CTO. Bifemoral 7F arterial access was obtained, with cannulation of the left coronary system and the RCA. HDR (hydrodynamic plaque recanalization) was used as the upfront antegrade technique. Next, we advanced with a polymeric guidewire and failed to cross the lesion. J-CTO score was evaluated as 3—very difficult. The strategy was switched to a retrograde approach with reverse CART (Controlled Antegrade and Retrograde Tracking). Tip-in of the retrograde, intralesional microcatheter positioning was achieved, and a follow-me technique was used to seat the antegrade microcatheter in the distal part of the RCA. The lesion was dilated with noncompliant and cutting balloons and stented with three DESs. Control angiography revealed good stent expansion, but the presence of a distal subintimal hematoma with flow impairment, which was decompressed using the ‘Cuttering technique’. After we injected contrast into the guide extension catheter, extensive subintimal contrast staining and hydraulic dissection suggestive of a contained perforation were observed. The complication was successfully managed with additional stent implantation, without hemodynamic instability or pericardial effusion. The final result was satisfactory with TIMI III flow and an MBG (myocardial blush grade) of 3 (Figure 1). The main steps of the procedure are described in Figure 2. At discharge, the patient was stable and asymptomatic.

**Figure 3:**
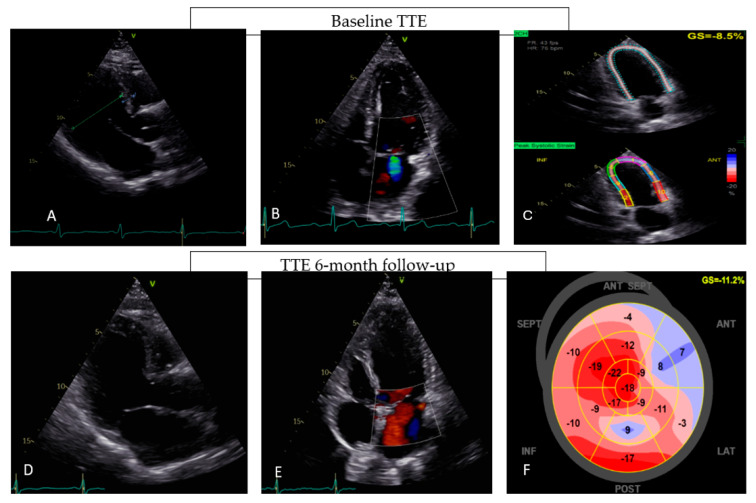
Baseline and 6-month follow-up TTE. Legend: (**A**). Parasternal view, enlarged left ventricle (62 mm); (**B**) apical 4-chamber view, mild mitral regurgitation; (**C**) global longitudinal strain 2 chamber; (**D**) parasternal view (at 6 months), modest improvement in ventricular geometry; (**E**) apical 4-chamber view showing no mitral regurgitation; (**F**) bull’s eye global longitudinal strain evaluation.

After 6 months of follow-up, the patient presented clinically stable, showing no recurrent episodes of angina, HF symptoms, or hospitalizations. Functional status improved from NYHA class III at baseline to NYHA class II. Quality of life assessed by MLHFQ showed a significant clinical improvement with a total score decreasing from 56 to 32 at follow-up. Repeat TTE demonstrated a modest improvement of LVEF (from 37% to 42%) and GLS (global longitudinal strain) (from −8.5% to −11.2%), a reduction in LV end-diastolic volume, a moderate improvement in diastolic function, and a regression of functional ischemic mitral regurgitation (Figure 3). Regarding laboratory examination, a decrease in NT-proBNP levels (up to 1430) was recorded. During follow-up, no late procedural complications or adverse events were observed. GDMT for HF was maintained, and dual antiplatelet therapy was continued without bleeding complications.

At the 12-month follow-up, the patient presented with NYHA class II, MLHFQ decreased from 32 to 20, NT-proBNP was 893 pg/mL, and LVEF 43%, measured with Simpson’s rule with a slight decrease in LV volumes and filling pressures (mild decrease in TR (tricuspid regurgitation) velocity, reduction in LAVI (left atrial volume indexed), and a progressive shortening of IVRT and DT) (Table 2). The patient was encouraged to continue OMT for ischemic HF. Table 3 summarizes the patient’s clinical and laboratory characteristics at baseline and at 6- and 12-month follow-ups.

### 3.2. Case 2

A 66-year-old man from a rural area, a former smoker with arterial hypertension, severe chronic obstructive pulmonary disease (GOLD stage IV), and obesity (BMI 33.98 kg/m^2^), was admitted for recurrent dyspnea (NYHA class III) that had progressed over the previous weeks despite OMT. His MLHFQ score was 66. In October 2024, he had presented to another hospital with exertional angina (Canadian Cardiovascular Society class III), and coronary angiography at that time revealed chronic total occlusions of both the RCA and the LAD artery.

Electrocardiography showed Q waves in the inferior leads and across V1–V6. TTE demonstrated reduced LV systolic function (LVEF 37%), biventricular dilatation, moderate tricuspid regurgitation, diastolic dysfunction (increased LAVI, markedly elevated TR velocity), and an estimated pulmonary artery systolic pressure of 40 mmHg, suggestive of associated pulmonary hypertension (Table 1). Laboratory evaluation revealed normal glycemia and optimally controlled lipid levels (LDL cholesterol 50 mg/dL), preserved renal function (creatinine 0.99 mg/dL), non-elevated high-sensitivity troponin, and an NT-proBNP level of 4500 pg/mL.

The patient remained symptomatic despite ongoing medical therapy for ischemia and HF comprising dual antiplatelet therapy, beta-blocker, angiotensin-converting enzyme inhibitor, statin, mineralocorticoid receptor antagonist (spironolactone), SGLT2 inhibitors, loop diuretic, and trimetazidine.

**Figure 4:**
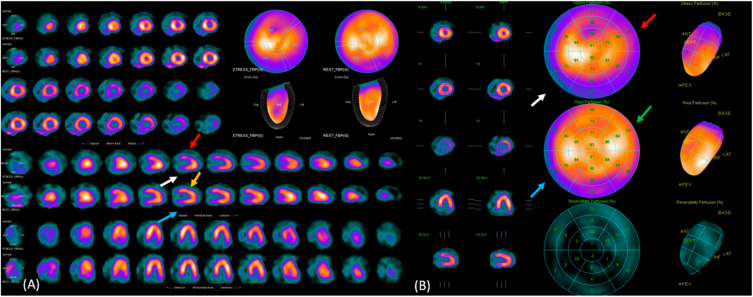
Myocardial perfusion SPECT demonstrating viable myocardium in LAD and RCA territories. (**A**) Stress images (short-axis, vertical long-axis, and horizontal long-axis views) showing partial perfusion defects in the anterior wall (red arrow) and obvious ischemia in the inferior wall (white arrow). Rest images demonstrated reversible ischemia in the anterior wall (yellow arrow) and partial reversible ischemia in the inferior territory (blue arrow). (**B**) Corresponding stress, rest, and reversibility polar maps demonstrated inducible ischemia in both vascular distributions, especially in the LAD territory (red and green arrows) and moderate reversible perfusion in the RCA territory (white and blue arrows), consistent with viable myocardium.

Myocardial perfusion SPECT (rest and stress acquisitions) was subsequently obtained to identify the viable myocardium and demonstrated preserved tracer uptake in both the inferior and anterior territories, suggesting potentially viable myocardium that could support revascularization consideration. The rest acquisitions demonstrated sustained radiotracer retention corresponding to hibernating myocardium, while the stress images showed inducible ischemia in the same segments. Figure 4 illustrates the SPECT findings, rest and stress perfusion acquisitions, and segmental analysis, supplemented by explanatory labels that highlight viable and ischemic territories.

**Figure 5:**
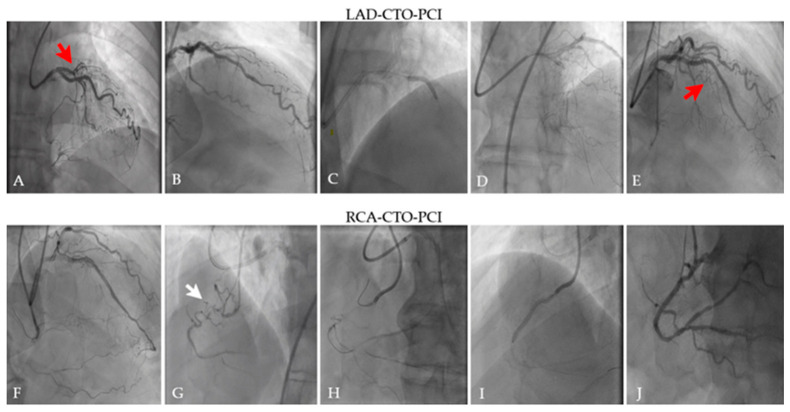
Percutaneous recanalization of CTO in the LAD and RCA. Legend: LAD—left anterior descending, CTO—chronic total occlusion, RCA—right coronary artery. (**A**) Diagnostic coronary angiography, LAD-CTO (red arrow); (**B**–**D**) antegrade guidewire advancement, crossing CTO, pre-dilatations with balloons, and stenting; (**E**) final result (red arrow); (**F**,**G**) diagnostic coronary angiography, collaterals supplying RCA, RCA-CTO (white arrow); (**H**,**I**) guidewire crossing, balloons pre-dilatations, stenting; (**J**) final result.

Based on imaging findings, persistent symptoms, and coronary angiography results, staged CTO-PCI of both the LAD and RCA was performed (Figure 5).

**Figure 6:**
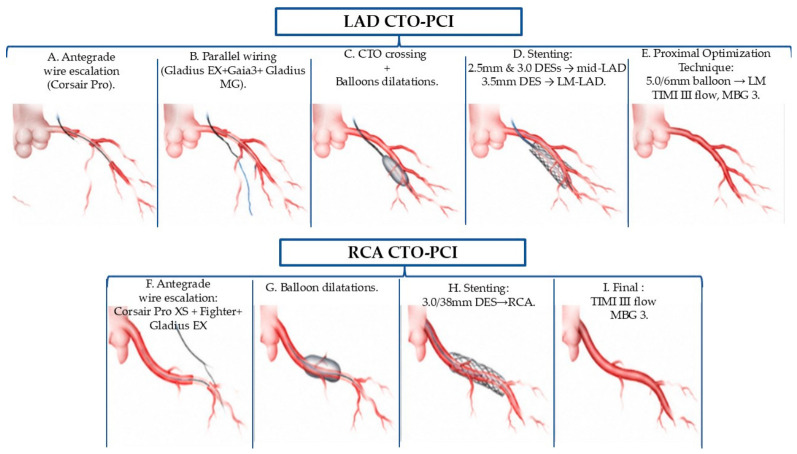
LAD/RCA CTO-PCI step by step. Legend: CTO—chronic total occlusion, DES—drug-eluting stents, LAD—left anterior descending, LM—left main, RCA—right coronary artery, TIMI—Thrombolysis in Myocardial Infarction, MBG—Myocardial Blush Grade.

Staged CTO-PCI of LAD and RCA was performed using radial and femoral access with contemporary antegrade wire escalation techniques. A right-dominant coronary arterial system was identified with 50% LM Medina type 1.1.0, 50–70% ostial LAD stenosis, followed by a CTO in the mid-LAD (J-CTO 4), and the RCA (J-CTO 2) was chronically occluded in the proximal segment, both occlusions with collateral filling. LAD-CTO was managed via antegrade wire escalation (AWE technique) followed by parallel over a recross dual-lumen, obtaining successful crossing. After balloon dilatations, two 2.5 mm and 3.0 DESs were implanted in the mid-LAD, and a DES across LM-LAD, followed by POT (proximal optimization technique) in LM with a good result (TIMI III, MBG 3). Thereafter, the RCA CTO was managed using AWE following pre-dilatation with a balloon; a DES was implanted with an optimal angiographic result (TIMI III, MBG 3) without major complications. The steps of the procedures are depicted in Figure 6. Three days after the procedure, the patient was stable, event-free, and discharged on OMT for ischemic HF.

**Figure 7:**
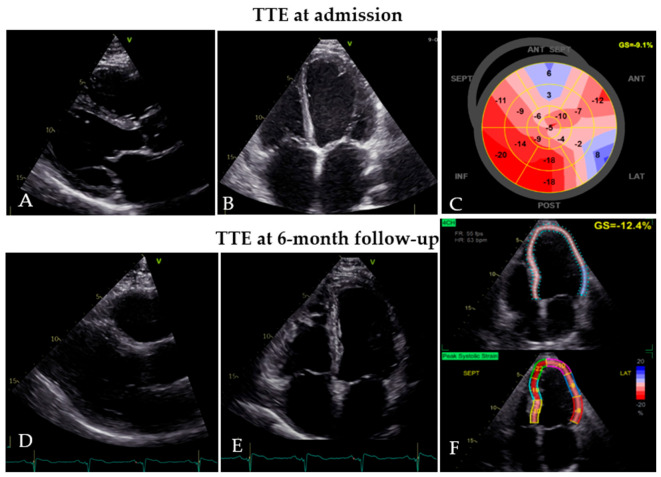
TTE at baseline and 6-month follow-up. (**A**) Parasternal view with LV dimension measurement; (**B**) apical 4-chamber view; (**C**) baseline global longitudinal strain bull’s-eye; (**D**) parasternal view at 6 months with slightly improved LV dimension; (**E**) apical 4-chamber view; (**F**) follow-up strain evaluation showing a modest recovery.

At 6 months, objective examination showed no signs of HF. The patient reported a clear improvement in functional status (from NYHA class III to NYHA class II), no recurrent episodes of angina, and no other hospitalizations. Health-related quality of life improved considerably, and the MLHFQ score decreased from 66 at baseline to 32. NT-proBNP was also significantly reduced from 4500 pg/mL to 2188 pg/mL. TTE demonstrated partial reverse remodeling (improvement in LVEF and GLS, reduction in end-diastolic volume) and a reduction in right ventricular dimensions. Tricuspid regurgitation was quantified as II/III degree, and pulmonary artery systolic pressure showed a modest decrease (Figure 7). A progressive improvement in diastolic parameters was demonstrated (decrease in LAVI, shortening of IVRT and DT, reduction in TR velocity) (Table 2). The patient continued the prescribed medical therapy without complications, and no stent-related issues or late adverse events were observed during follow-up. At 12 months, clinical status improved to NYHA class I, the MLHFQ score decreased to 21, and NT-proBNP declined to 750 pg/mL. Although TTE showed a modest improvement in LVEF to 40%, there was a notable progress in myocardial relaxation, and lower LV filling pressures were demonstrated. Table 3 summarizes the patient’s baseline and 6- and 12-month follow-up characteristics. Conservative medical management was maintained.

### 3.3. Case 3

A 70-year-old man from an urban area, a former smoker with a history of recurrent HF decompensation (pulmonary edema), dilated ischemic cardiomyopathy (LAD PCI and LCx-PCI in 2024, and CTO of the RCA), diabetes mellitus, arterial hypertension, and chronic kidney disease, presented with worsening HF characterized by NYHA class III dyspnea and peripheral edema that had progressed over three weeks and deteriorated in the preceding two days. MLHFQ score was 84.

Electrocardiography showed sinus rhythm with left ventricular hypertrophy and T-wave inversion in the lateral leads. TTE demonstrated a dilated cardiomyopathy with reduced ejection fraction (35%), moderate mitral regurgitation, regional wall-motion abnormalities involving the septal and inferior left ventricular walls, and a significant impairment of diastolic function (Table 1). Laboratory evaluation revealed elevated NT-proBNP (3059 pg/mL), normal troponin, increased C-reactive protein (22 mg/L), poorly controlled LDL cholesterol (145 mg/dL), preserved renal function, and mildly elevated glucose (135 mg/dL).

At admission, the patient received GDMT for ischemic HF, including dual antiplatelet therapy (aspirin and ticagrelor), a high-intensity statin (atorvastatin), a beta-blocker, ARNI, a potassium-sparing diuretic, a loop diuretic, and an SGLT2i, along with strict lifestyle and dietary recommendations.

Diagnostic coronary angiography revealed a 75–90% calcific stenosis on the mid RCA, followed by a CTO in the distal segment supplied by collaterals from the left system. SPECT was performed, using both rest and stress acquisitions, which showed preserved tracer uptake in the inferior territory of the LV, indicating the presence of viable myocardium with inducible ischemia.

**Figure 8:**
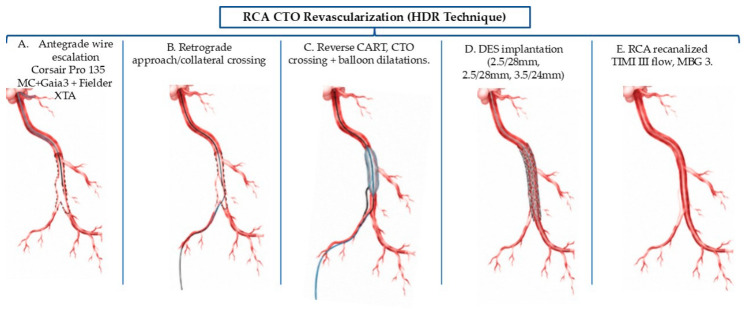
RCA CTO-PCI step by step. Legend: RCA—right coronary artery, DES—drug-eluting stents, TIMI—Thrombolysis in Myocardial Infarction, MBG—Myocardial Blush Grade, HDR—Hydrodynamic Contrast Recanalization.

**Figure 9:**
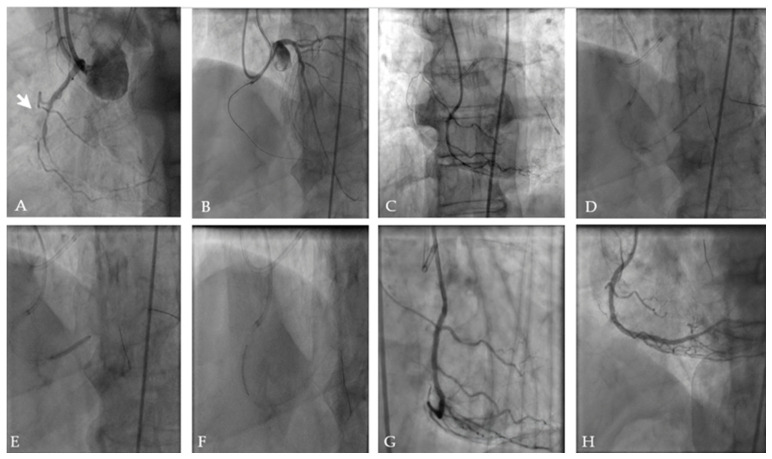
Percutaneous revascularization of a right coronary artery chronic total occlusion. Legend: (**A**) Diagnostic angiography showing RCA-CTO (white arrow); (**B**–**F**) CTO crossing using antegrade techniques with guidewire, microcatheter support, and stenting; (**G**,**H**) final angiographic result showing successful revascularization with restoration of TIMI 3 flow.

Given persistent symptoms despite OMT and evidence of viable myocardium in the RCA territory, CTO-PCI was conducted. J-CTO score was 3. RCA recanalization was performed via right radial and right femoral access. The HDR technique was conducted, and because of deep intubation of the guide catheter, the patient developed ventricular fibrillation, needing resuscitation (after 200J, sinus rhythm was restored). The guide catheter was switched, and the lesion was recrossed. Following balloon pre-dilatation, 3 DESs were implanted with an optimal final angiographic result with TIMI III flow and MBG 3. Figure 8 and Figure 9 illustrate the revascularization technique.

After the procedure, the patient remained hemodynamically stable without complications and was discharged after five days on guideline-directed OMT. Ezetimibe was added to high-intensity statin therapy.

**Figure 10:**
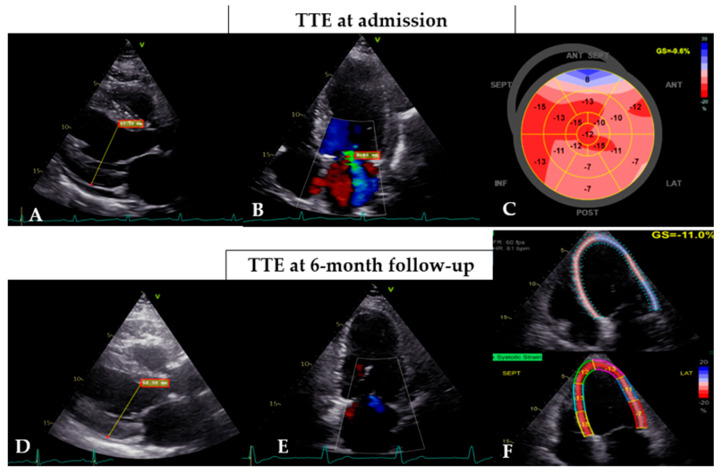
Baseline and 6-month follow-up TTE. Legend: TTE = transthoracic echocardiography. (**A**) Parasternal view with LV dimension measurement in diastole (63.18 mm); (**B**) apical 4-chamber view, moderate mitral regurgitation with a vena contracta width of approximately 6 mm; (**C**) baseline global longitudinal strain (GLS) bull’s-eye; (**D**) parasternal view at 6 months with improved LV dimension; (**E**) apical 4-chamber view, mild mitral regurgitation; (**F**) follow-up strain evaluation showing partial recovery.

At the 6-month follow-up, significant clinical improvement was observed, with resolution of peripheral edema and improvement in functional status from NYHA class III to class I–II. No recurrent angina or HF-related hospitalization was noted. The levels of NT-proBNP diminished from 3059 pg/mL to 1260 pg/mL, and clinical stabilization was noticed. MLHFQ score showed a decline from 84 points to 31 points. TTE revealed a modest improvement in left ventricular function, a minor mitral regurgitation, and better diastolic parameters (shortening of DT and IVRT, modest reduction in LAVI) (Figure 10). No arrhythmic events or late stent-related complications were seen during follow-up.

At the 12-month follow-up, the patient improved to NYHA class I and was asymptomatic at evaluation. The MLHFQ score decreased to 10, NT-proBNP was near normal at 502 pg/mL, and TTE showed normalized left ventricular EF (50% by Simpson’s method), normal chamber volumes, and a substantial improvement in diastolic function. Table 2 and Table 3 summarize the patient’s baseline and 6- and 12-month follow-up characteristics.

## 4. Discussion

The management of CTOs in patients with ischemic HFrEF remains a grey area, as randomized trials have not demonstrated reductions in mortality or major adverse cardiovascular events (MACE) following CTO-PCI [5,6,7,28]. These studies enrolled a limited number of high-risk patients with advanced HF, severely impaired systolic function, recurrent decompensations, and complex coronary anatomy. Therefore, the applicability of their findings to real-world ischemic HFrEF populations remains limited. In addition, myocardial viability assessment was not systematically incorporated into patient selection, potentially attenuating the observed benefit of revascularization in patients with predominantly scarred myocardium [29,30].

In the present series, all patients demonstrated viable myocardium within the CTO-related territory on SPECT imaging, which strongly influenced the revascularization strategy. Viability assessment was based on preserved radiotracer uptake >50% combined with inducible ischemia on stress-rest SPECT imaging. These criteria were previously associated with a greater likelihood of functional recovery after revascularization [14]. Despite only modest improvement in global EF, all three patients experienced meaningful symptomatic and clinical benefit, including improvement in NYHA functional class, reductions in NT-proBNP levels and MLHFQ scores, and greater clinical stability, without HF-related rehospitalizations during follow-up. These findings were consistent with previous studies suggesting that the principal benefit of CTO-PCI in ischemic HFrEF may derive from the reduction in ischemic burden and improved myocardial efficiency rather than from major recovery of systolic function alone [31,32,33,34,35].

An important observation in our cases was the improvement in diastolic function after revascularization. Chronic ischemia and hibernating myocardium impaired myocardial relaxation and increased ventricular stiffness, ultimately contributing to elevated filling pressures and exertional dyspnea [36,37,38]. By restoring coronary blood flow, revascularization may enhance myocardial energetics, calcium handling, and lusitropic function. This may reduce diastolic wall stress and improve symptoms independently of substantial EF recovery [39,40].

Although modest improvements in systolic and diastolic function were observed after CTO-PCI, the predominant gains were symptomatic. Relief of chronic ischemia in viable myocardium was associated with symptomatic and functional improvement. Accordingly, EF alone may not fully reflect the clinical response after CTO-PCI.

Nevertheless, these observations should be interpreted within the context of the current evidence base, in which a definitive mortality benefit after CTO-PCI has not been consistently demonstrated. Although observational studies and registries suggest a greater clinical stability following revascularization, randomized trials have not consistently demonstrated reductions in mortality or MACE. Likewise, the long-term prognostic benefit of CTO-PCI in patients with ischemic HFrEF remains uncertain, particularly regarding survival and ventricular remodeling. Therefore, the findings of the present case series should be considered as exploratory and hypothesis-generating.

Another key aspect highlighted by this case series is the procedural feasibility of contemporary CTO-PCI techniques in high-risk patients when performed in experienced centers. All lesions were complex (moderate to high J-CTO scores) and required advanced crossing strategies, yet procedural success was achieved in all cases without major long-term adverse consequences during follow-up. These findings are consistent with data from contemporary studies and registries demonstrating improved CTO-PCI success rates and procedural safety using modern techniques and dedicated devices [5,41].

Interpretation of symptomatic and biomarker improvements in the present cases should be made with caution. All patients continued GMDT during follow-up. For that reason, the relative contributions of CTO-PCI and ongoing OMT cannot be fully separated in this observational case series. Furthermore, placebo effects, repeated clinical examinations during follow-up, regression to the mean, and improved treatment adherence may also have contributed to the observed symptomatic improvement. At the same time, all patients remained symptomatic despite established OMT before PCI, and no major changes in pharmacotherapy occurred during follow-up. The temporal association between successful CTO-PCI and subsequent improvement in symptoms, NT-proBNP levels, and quality of life suggests a potential contribution of ischemia relief in these selected patients.

Various limitations should be acknowledged. This is a small observational case series without a control group, limiting generalizability and precluding causal inference. Follow-up duration was relatively short, and improvements may partially reflect optimization of GDMT. The observational design does not allow differentiation between the effects of CTO-PCI and continued GDMT during follow-up. Larger prospective studies are needed to clarify the role of viability-guided CTO-PCI in patients with ischemic HFrEF.

Taken together, these findings raise the possibility that, in ischemic HFrEF, the primary benefit of CTO-PCI may derive from a reduction in ischemic burden and subsequent symptomatic and functional improvement rather than recovery of contractile function. However, these observations remain exploratory and require validation in larger prospective studies.

## 5. Conclusions

In carefully selected high-risk patients with ischemic HFrEF and demonstrable myocardial viability, CTO revascularization using contemporary techniques was associated with symptomatic and functional improvement when added to GDMT in these selected patients. Even in the absence of substantial recovery of global systolic function, a reduction in ischemic burden may contribute to improved clinical status and quality of life. Given the small observational nature of this case series, these findings should be interpreted cautiously and considered hypothesis-generating. Further prospective studies and randomized viability-guided investigations are required to better define the long-term clinical and prognostic impact of CTO-PCI in patients with ischemic HFrEF.

## Figures and Tables

**Table 1:** Baseline echocardiographic parameters.

Echocardiographic Parameters	Case 1	Case 2	Case 3
End-systolic Diameter (mm)	40	45	48
End-diastolic Diameter (mm)	59	63	60
End-systolic Volume (mL)	105	100	94
End-diastolic Volume (mL)	167	159	165
CO at 80 bpm	3.8	2.5	2.9
LVEF with Simpson’s method (%)	37	37	35
Global longitudinal strain (%)	−8.5	−9.1	−9.6
Mitral annular plane systolic excursion (mm)	8	8	9
E/A	0.9	1	1
E (cm/s)	85	95	86
e’ (cm/s)	5	5	6
E/e’ (cm/s)	17	19	14.3
Left atrial volume indexed (mL/m^2^)	34	40	35
Tricuspid regurgitation velocity (m/s)	2	3.8	1.9
Isovolumic relaxation time (ms)	112	135	167
Deceleration time (ms)	320	300	298

Legend: ms—milliseconds, m/s—meters per second, mL/m^2^—milliliters per square meter, cm/s—centimeters per second, mm—millimeters, mL—milliliters. LVEF—left ventricular Ejection Fraction, CO—cardiac output at 80 bpm was calculated using echocardiographic stroke volume derived from LVOT cross-sectional area and LVOT velocity-time integral (VTI), standardized to a heart rate of 80 beats/min.

**Table 2:** Changes in echocardiographic parameters during follow-up.

TTE Parameter	Case 1 (6 mo)	Case 1 (12 mo)	Case 2 (6 mo)	Case 2 (12 mo)	Case 3 (6 mo)	Case 3 (12 mo)
LVEF (%)	42	43	39	40	40	50
E/A	0.98	1.1	0.97	1	0.96	1.05
E/e’ (cm/s)	11	12	14	13	12	11
DT (ms)	285	256	267	234	269	242
IVRT (ms)	103	95	119	106	141	115
LAVI (mL/m^2^)	33	29	36	34	33	31
TR velocity (m/s)	1.8	1.7	3	2.7	1.8	1.74

Legend: LVEF—Left Ventricular ejection fraction, DT—deceleration time, IVRT—Isovolumic Relaxation Time, LAVI—Left Atrial Volume Index, TR—tricuspid regurgitation, mo—months, ms—milliseconds, m/s—meters per second, mL/m^2^—milliliters per square meter, cm/s—centimeters per second.

**Table 3:** Clinical, imaging, and biomarker changes from baseline to 6- and 12-month follow-ups after CTO-PCI.

Parameter	Case 1	Case 2	Case 3
Age/Sex	74/F	66/M	70/M
Target CTO vessel	RCA	LAD + RCA	RCA
Viable myocardium	+	+	+
JCTO score	3	LAD 4/RCA 2	3
Baseline NYHA class	III	III	III
6 mo NYHA class	II	II	I/II
12 mo NYHA class	II	I	I
TIMI flow/MBG	III/3	III/3	III/3
Baseline MLHFQ score	56	66	84
6 mo MLHFQ score	32	32	31
12 mo MLHFQ score	20	21	10
Baseline NT-proBNP	3570	4500	3059
6 mo NT-proBNP	1430	2188	1260
12 mo NT-proBNP	893	750	502
HF hospitalizations during follow-up	0	0	0
Periprocedural complications	Subintimal hematoma, Coronary dissection	None	VF

Legend: CTO—chronic total occlusion, NYHA—New York Heart Association, TIMI—Thrombolysis in Myocardial Infarction, MBG—Myocardial Blush Grade, MLHFQ—Minnesota Living with Heart Failure Questionnaire, NT—proBNP-N-terminal pro–B-type Natriuretic Peptide, HF—heart failure, mo—months, VF—ventricular fibrillation.

## Data Availability

Data are available from the last author upon request.

## Abbreviations

The following abbreviations are used in this manuscript:

CTO	Chronic total occlusion
HFrEF	Heart failure with reduced ejection fraction
SPECT	Single-photon emission computed tomography
GDMT	Guideline-directed medical therapy
NYHA	New York Heart Association
NT-proBNP	N-terminal pro-brain natriuretic peptide
MLHFQ	Minnesota Living with Heart Failure Questionnaire
HF	Heart failure
PCI	Percutaneous coronary intervention
LV	Left ventricular
EF	Ejection fraction
CAD	Coronary artery disease
ARNI	Angiotensin Neprilysin Inhibitor
ACEI	Angiotensin converting-enzyme inhibitor
ARB	Angiotensin II Receptor Blocker
SGLT2i	sodium-glucose cotransporter-2 inhibitor
OMT	Optimal medical therapy
TIMI	Thrombolysis In Myocardial Infarction
MBG	Myocardial blush grading
MACE	Major adverse cardiovascular events
LAD	Left anterior descending
LCx	Circumflex artery
RCA	Right coronary artery
IVRT	Isovolumic Relaxation Time
TTE	Transthoracic echocardiography
LAVI	Left Atrial Volume Index
DT	Deceleration time
TR	Tricuspid regurgitation
DES	Drug-eluting stents
MACE	Major adverse cardiac events
VF	Ventricular fibrillation
GLS	Global longitudinal strain
HDR	Hydrodynamic contrast recanalization
